# Silver Nanoparticles as Potential Antibacterial Agents

**DOI:** 10.3390/molecules20058856

**Published:** 2015-05-18

**Authors:** Gianluigi Franci, Annarita Falanga, Stefania Galdiero, Luciana Palomba, Mahendra Rai, Giancarlo Morelli, Massimiliano Galdiero

**Affiliations:** 1 Dipartimento di Biochimica, Biofisica e Patologia Generale, Seconda Università degli Studi di Napoli, Vico L. De Crecchio 7, 80138 Napoli, Italy; E-Mail: gianluigi.franci@unina2.it; 2 Department of Pharmacy, University of Naples Federico II, 80100 Naples, Italy; E-Mails: annarita.falanga@unina.it (A.F.); stefania.galdiero@unina.it (S.G.); giancarlo.morelli@unina.it (G.M.); 3 Istituto di Biostrutture e Bioimmagini, CNR, 80100 Napoli, Italy; 4 CIRPEB, and DFM, University of Naples Federico II, 80100 Naples, Italy; 5 Department of Experimental Medicine, II University of Naples, 80138 Naples, Italy; E-Mail: luciana.palomba@igb.cnr.it; 6 Department of Biotechnology, Sant Gadge Baba Amravati University, Amravati, Maharashtra 444602, India; E-Mail: mkrai123@rediffmail.com

**Keywords:** AgNPs, antibacterial, resistence, biofilm

## Abstract

Multi-drug resistance is a growing problem in the treatment of infectious diseases and the widespread use of broad-spectrum antibiotics has produced antibiotic resistance for many human bacterial pathogens. Advances in nanotechnology have opened new horizons in nanomedicine, allowing the synthesis of nanoparticles that can be assembled into complex architectures. Novel studies and technologies are devoted to understanding the mechanisms of disease for the design of new drugs, but unfortunately infectious diseases continue to be a major health burden worldwide. Since ancient times, silver was known for its anti-bacterial effects and for centuries it has been used for prevention and control of disparate infections. Currently nanotechnology and nanomaterials are fully integrated in common applications and objects that we use every day. In addition, the silver nanoparticles are attracting much interest because of their potent antibacterial activity. Many studies have also shown an important activity of silver nanoparticles against bacterial biofilms. This review aims to summarize the emerging efforts to address current challenges and solutions in the treatment of infectious diseases, particularly the use of nanosilver antimicrobials.

## 1. Introduction

Increasing hospital and community-acquired infections due to bacterial multidrug-resistant (MDR) pathogens for which current antibiotic therapies are not effective represent a growing problem. Antimicrobial resistance is, thus, one of the major threats to human health [1], since it determines an increase of morbidity and mortality as a consequence of the most common bacterial diseases [2]. Resistance genes have recently emerged [3], favoured by improper use of antibiotics [4]; hence, the first step in combating resistance envisions the reduction of antibiotic consumption [5]. Antimicrobial resistance is a complex mechanism whose etiology depends on the individual, the bacterial strains and resistance mechanisms that are developed [6]. The emergence of resistance against newly developed antibiotics [7], further supports the need for innovation, monitoring of antibiotic consumption, prevention, diagnosis and rapid reduction in the misuse of these drugs. It is thus necessary to optimize antibiotics’ pharmacokinetics and pharmacodynamics in order to improve treatment outcomes and reduce the toxicity and the risk of developing resistance [8]. To address the problem of resistance, it will be necessary to change the protocols of use of antimicrobials so that these drugs are administered only when all other treatment options have failed [4]; and joint efforts of governments and academic networks are needed to fight against the globally spreading of multidrug resistant pathogens. Today, there is a need to seek alternative treatments [9]. Non-traditional antibacterial agents are thus of great interest to overcome resistance that develops from several pathogenic microorganisms against most of the commonly used antibiotics [4].

## 2. Silver Nanoparticles and Antibacterial Activity

Nanoparticles are now considered a viable alternative to antibiotics and seem to have a high potential to solve the problem of the emergence of bacterial multidrug resistance [10]. In particular, silver nanoparticles (AgNPs) have attracted much attention in the scientific field [11,12,13]. Silver has always been used against various diseases; in the past it found use as an antiseptic and antimicrobial against Gram-positive and Gram-negative bacteria [14,15,16] due to its low cytotoxicity [17]. AgNPs were considered, in recent years, particularly attractive for the production of a new class of antimicrobials [4,18,19,20,21,22,23,24,25] opening up a completely new way to combat a wide range of bacterial pathogens. Although the highly antibacterial effect of AgNPs has been widely described, their mechanism of action is yet to be fully elucidated. In fact, the potent antibacterial and broad-spectrum activity against morphologically and metabolically different microorganisms seems to be correlated with a multifaceted mechanism by which nanoparticles interact with microbes. Moreover, their particular structure and the different modes of establishing an interaction with bacterial surfaces may offer a unique and under probed antibacterial mechanism to exploit. From a structural point of view, AgNPs have at least one dimension in the range from 1 to 100 nm and more importantly, as particle size decreases, the surface area-to-volume ratio greatly increases. As a consequence, the physical, chemical and biological properties are markedly different from those of the bulk material of origin. Several mechanisms of action have been proposed by different authors, and the most corroborated are described below and in Table 1 [4].

**Table 1:** Details of AgNPs and their mechanisms of action against bacteria and biofilms.

Bacteria	Mechanism of Action	References
*Acinetobacter baumannii*	Alteration of cell wall and cytoplasm.	[26,27]
*Escherichia coli*	Alteration of membrane permeability and respiration	[26,28,29,30,31,32,33,34,35,36,37,38,39,40,41,42,43,44]
*Enterococcus faecalis*	Alteration of cell wall and cytoplasm.	[42,45,46]
*Klebsiella pneumoniae*	Alteration of membrane	[28,41,47]
*Listeria monocytogenes*	Morphological changes, separation of the cytoplasmic membrane from the cell wall, plasmolysis	[47]
*Micrococcus luteus*	Alteration of membrane	[28]
Nitrifying bacteria	inhibits respiratory activity	[31]
*Pseudomonas aeruginosa*	Irreversible damage on bacterial cells; Alteration of membrane permeability and respiration	[17,28,32,33,36,41,42,43,44,48,49,50]
*Proteus mirabilis*	Alteration of cell wall and cytoplasm.	[43,44]
*Staphylococcus aureus*	Irreversible damage on bacterial cells	[17,26,31,34,37,39,40,41,48,51,52]
*Staphylococcus epidermidis*	Inhibition of bacterial DNA replication, bacterial cytoplasm membranes damage, modification of intracellular ATP levels	[36,52]
*Salmonella typhi*	Inhibition of bacterial DNA replication, bacterial cytoplasm membranes damage, modification of intracellular ATP levels	[33,36,48,51]
*Vibrio cholerae*	Alteration of membrane permeability and respiration	[33]

**Figure 1:**
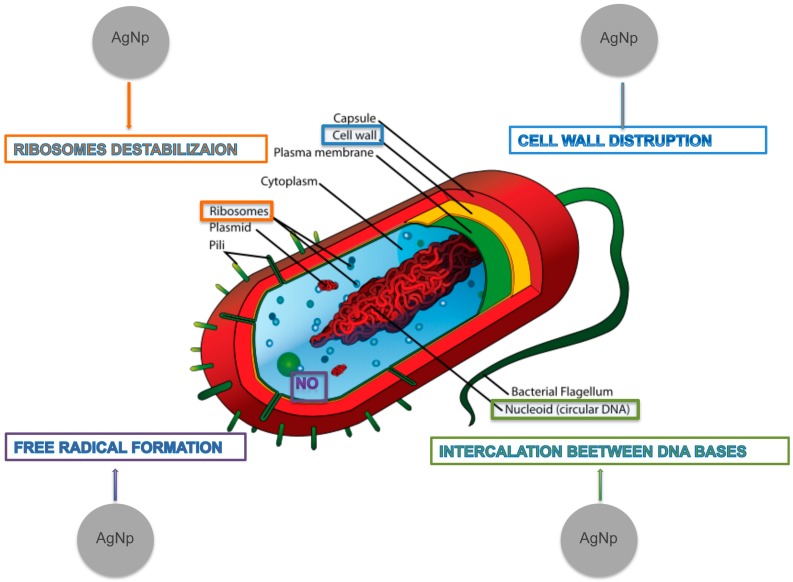
Mechanisms of AgNPs’ toxic action.

AgNPs are able to physically interact with the cell surface of various bacteria. This is particularly important in the case of Gram-negative bacteria where numerous studies have observed the adhesion and accumulation of AgNPs to the bacterial surface. Many studies have reported that AgNPs can damage cell membranes leading to structural changes, which render bacteria more permeable [14,53]. This effect is highly influenced by the nanoparticles’ size, shape and concentration [53,54,55,56] and a study using *Escherichia coli* [14] confirmed that AgNPs accumulation on the membrane cell creates gaps in the integrity of the bilayer which predisposes it to a permeability increase and finally bacterial cell death [19]. Several studies have shown that AgNP activity is strongly dependent on the size [46,47]. In fact, the bactericidal activity of AgNPs of smaller dimensions (<30 nm) was found to be optimal against *Staphylococcus aureus* and *Klebsiella pneumoniae* [49]. Smaller nanoparticles seem to have a superior ability to penetrate into bacteria. In fact, the interactions with the membranes and any resulting damage, which may lead to cell death, are certainly more evident in the case of nanoparticles with smaller diameter and a positive zeta potential. Electrostatic forces that develop when nanoparticles with a positive zeta potential encounter bacteria with a negative surface charge promote a closer attraction and interaction between the two entities and possibly the penetration in bacterial membranes. Indeed, the zeta potential along with the size of the nanoparticles is a fundamental parameter for controlling the antimicrobial activity and more effective nanoparticles have a positive zeta potential and a reduced size. As said earlier, AgNPs have a surface/volume ratio much greater than the corresponding bulk material; therefore, modalities and amount of the interactions with the bacterial surfaces are facilitated and determine a higher antibacterial activity. One should also consider that a certain amount of cationic silver is released from the nanoparticles when these are dissolved in water or when they penetrate into the cells. In effect, nanoparticles have a higher antibacterial activity than the free ions of silver, whereby the antibacterial properties are attributed to both the physical properties of nanoparticles and the elution of silver ions [57]. It is likely that a combined effect between the activity of the nanoparticles and free ions contributes in different ways to produce a strong antibacterial activity of broad spectrum. Furthermore, the fact that bacterial resistance to elemental silver is extremely rare [58] emphasizes the presence of multiple bactericidal mechanisms that act in synergy. The silver ions bind to the protein and nucleic acid negatively charged, causing structural changes and deformations in the wall, in the membranes and in the nucleic acids of the bacterial cell. In fact, silver ions interact with a number of electron donor functional groups such as thiols, phosphates, hydroxyls, imidazoles and indoles. The AgNPs also damage membranes and induce the release of reactive oxygen species (ROS), forming free radicals with a powerful bactericidal action [46]. Silver ions or small AgNPs can easily enter the microbial body causing the damage of its intracellular structures. As a consequence ribosomes may be denatured with inhibition of protein synthesis, as well as translation and transcription can be blocked by the binding with the genetic material of the bacterial cell [33,59,60]. Protein synthesis has been shown to be altered by treatment with AgNPs and proteomic data have shown an accumulation of immature precursors of membrane proteins resulting in destabilization of the composition of the outer membrane [61]. In Figure 1 we summarize the possible toxicity mechanisms of AgNPs.

The correlation between the bactericidal effect and AgNP concentrations is bacterial class dependent [22]. Indeed, *Pseudomonas aeruginosa* and *Vibrio cholera* were more resistant than *E. coli* and *Salmonella typhi*, but at concentrations above 75 μg/mL, the bacterial growth was completely abolished [50]. In this perspective, Kim *et al.* [23] studied AgNPs antimicrobial activity against *E. coli* and *S. aureus* showing that *E. coli* was inhibited at low concentrations, while the inhibitory effects on the growth of *S. aureus* were less marked [46]. AgNPs have been shown to be definitely an effective antibiotic against *E. coli*, *S. typhi*, *Staphylococcus epidermidis* and *S. aureus* [52]. Increasing scientific evidence has demonstrated that AgNP activity would depend not only on their concentration and size [16,41], but also on their shape [45]. In this regard, *E. coli* seems to respond better to triangular nanoparticles and is inhibited at low concentrations [46]. Pal *et al.* [35] studied the effect of nanoparticles with spherical, rod-like and triangular shapes against *E. coli*. They showed that all of them had antimicrobial activity, with the triangular nanoparticles being qualitatively more effective. Probably the triangular shape gives a greater positive charge to the nanoparticles, which together with the active facets on a triangular-shaped particle is able to ensure a greater activity. It has been suggested that AgNPs also interfere with bacterial replication processes by adhering to their nucleic acids [41]. This assumption, however, is controversial: for some authors AgNPs do not damage DNA [55], while according to others [56] they intercalate into the DNA. All factors which influence the activity of AgNPs (concentration, size, shape, UV radiation and the combination with various antibiotics) should be taken into consideration when preparing AgNPs for clinical use [20]. Notwithstanding the many conflicts in the literature regarding the effects of antibacterial AgNPs, it is likely that it is the result of a combined effect of each contributing feature, which provide a broad spectrum of antibacterial activity and decrease the probability of developing resistance [58].

**Figure 2:**
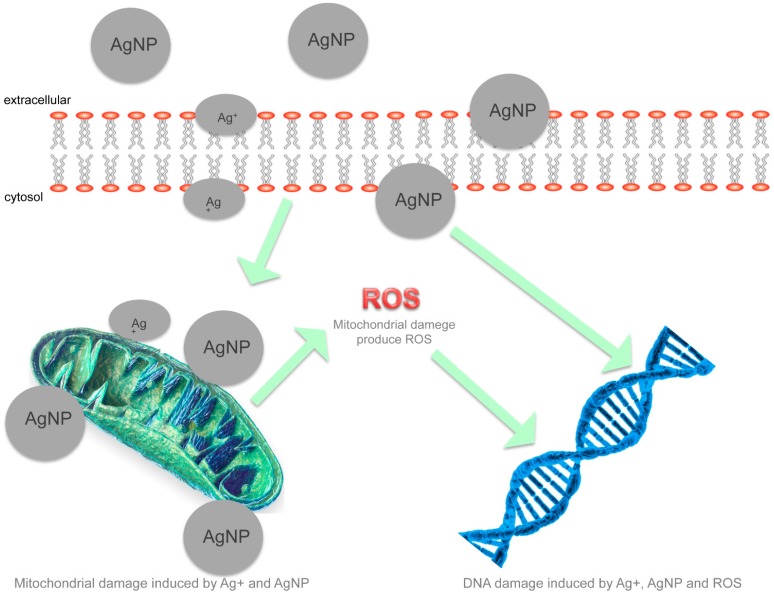
Schematic representation of various cellular responses to AgNP-induced toxicity mechanisms. In particular AgNPs induce mitochondrial and DNA damage by ROS.

In Figure 2, the hypothesized bactericidal mechanisms are reported, where mitochondrial and DNA damage through ROS is of particular interest and could be induced by AgNPs.

In light of the decreasing effectiveness of classical antibiotics due to the emergence of biological resistance, the use of AgNPs in association with antibiotic drugs can be seen as an alternative for such difficult treatments. In fact, Singh *et al.* [62] investigated individual and combined effects of AgNPs with 14 antibiotics belonging to seven classes against seven pathogenic bacteria using the disc-diffusion method. Their results showed the feasibility of the strategy, but different levels of activity increments, according to the class of antibiotic used, were observed. Aminoglycosides showed a small increase with the exception of gentamicin against *Acinetobacter baumannii* and kanamycin against *P. aeruginosa*. Considerable enhancement of the antibacterial effect was observed for amoxicillin in the presence of AgNPs against *P. aeruginosa* and penicillin demonstrated a 3-fold increase of efficiency against *Streptococcus mutans*. Vancomycin, with a 3.8-fold increase of activity against *Enterobacter aerogenes*, was reported to have the highest overall synergistic activity in combination with AgNPs compared to all other antibiotics. They also tested clinical derived bacterial strains, exhibiting resistance to one or more antibiotics belonging to the β-lactam class, and showed that the addition of AgNPs downsized MIC (minimum inhibitory concentration) into the susceptibility range, therefore, addition of AgNPs not only reduced MICs, but also rendered bacteria susceptible to antibiotic treatment. This is of great importance since the administration of small amounts of AgNPs in combination with antibiotics can reduce the required dose of antibiotics to achieve the same effect by up to 1000-fold. Synergistic action of AgNPs and antibiotics resulted in enhanced antibacterial effects; therefore, the simultaneous action of antibiotics and AgNPs can hamper the resistance development by pathogenic bacteria, also in view of the reduced amount of antibiotic administered. Fayaz *et al.* [63] suggested that the increase in synergistic effect may be caused by the bonding reaction between antibiotic and AgNPs. They tested a set of antibiotics and found that the highest percentage of fold increase was obtained with ampicillin followed by kanamycin, erythromycin and chloramphenicol against all test strains. Interestingly, they realised that the percentage of fold increase in ampicillin with AgNPs against Gram-positive and Gram-negative bacteria were almost identical, even though inhibition of Gram-positive bacteria is generally more difficult to obtain with AgNPs alone. Furthermore, a different study analysed a set of clinical bacterial isolates exhibiting resistance against conventional sulphonamide (trimethoprim) and glycopeptides (vancomycin) antibiotics [64]. A synergistic effect of antibiotics in conjugation with biologically synthesized AgNPs increased the susceptibility among the tested bacteria from 20% to 30%. The combined effect of AgNPs and antibiotics was notably against *E. coli*, *P. aeruginosa*, *S. aureus*, *K. pneumonia*, *Bacillus* spp, and *Micrococcus luteus*. These results are also in line with the findings reported by Birla *et al.* [65] who registered increasing efficiency of antibiotics like vancomycin, gentamycin, streptomycin, ampicillin and kanamycin when used in combination with AgNPs against *P. aeruginosa*, *S. aureus* and *E. coli*. Since AgNPs modified with different coatings such as polyethyleneimines [66], chitosan [48], glucosamine [67] and peptides (personal unpublished data) generally showed an increased antibacterial activity that has been related to the increased uptake as a consequence of a greater binding ability of nanoparticles to bacterial cells, Brown *et al.* [68] functionalised the surface of AgNPs with ampicillin (AgNP-AMP). They observed that AgNP-AMPs had increased biocidal activity compared to AgNPs. Their data suggested that the antimicrobial activity of functionalised AgNP-AMPs reside in the combined effect of the AgNP and the ampicillin carried on the surface of the nanoparticle.

The use of combination strategies for combating antibiotic resistance is slowly finding its way as a promising attempt to reduce the amount of antibiotics to be administered, therefore lowering the chances of steady resistance development. Selected studies on the antibacterial activity of AgNPs are summarized in Table 2.

**Table 2:** Selected studies on the antibacterial activity of silver nanoparticles.

Organism	Functionalization	Size (nm)	Effect	Ref.
*E. coli* *S. aureus*	unfunctionalized	Not declared	MIC 100 μg/mL	[4]
*E. coli*	unfunctionalized	10–15	MIC 25 μg/mL	[36]
*S. typhi*			MIC 25 μg/mL	
*S. aureus*			MIC 100 μg/mL	
*E. coli*	unfunctionalized	12	MIC_70_ 10 μg/mL	[32]
*E. coli* *S. aureus*	Unfunctionalized	13.5	MIC 3.3–6.6 nM MIC > 33 nM	[34]
*P. aeruginosa*	unfunctionalized	20–30	MIC 20 μg/mL	[69]
*E. coli* *V. cholerae* *S. typhi* *P. aeruginosa*	unfunctionalized	21	MIC 75 μg/mL	[33]
*E. coli* *S. aureus*	poly(amidehydroxyurethane)-coated	23	MIC 10 μg/mL	[37]
*Brucella abortus*	unfunctionalized	3–18	MIC 6–8 ppm	[70]
*E. coli*	citrate	30	MIC 5–10 μg/mL	[38]
*S. aureus*	unfunctionalized	5.5	MIC 0.2–4 μg/mL	[71]
*E. coli*	unfunctionalized	50	MIC_99_ 0.1 μg/mL	[35]
*E. coli* *S. aureus*	unfunctionalized	55	MIC 0.25 μg/mL	[40]
*V. cholerae* *ETEC*	unfunctionalized	88–100	MIC 1.6 × 10^5^ for mL MIC 1.2 × 10^6^ for mL	[72]

## 3. AgNPs Antibiofilm Activity

**Figure 3:**
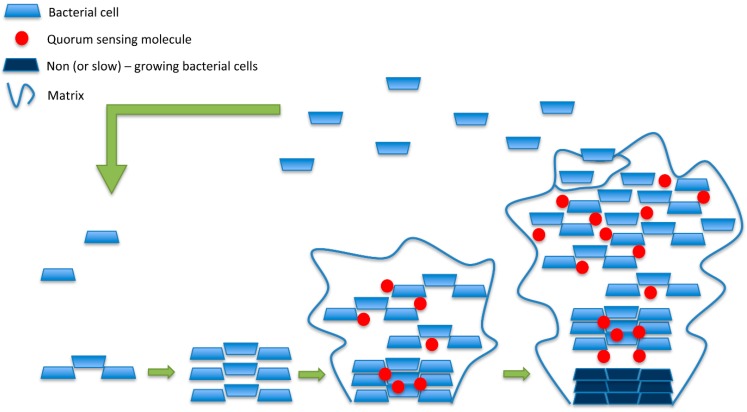
Planktonic cells adhere to the surface and proliferate. During biofilm maturation, the extracellular matrix and quorum sensing molecules are produced. Mature biofilms are generally characterized by an increased abundance of matrix materials, slow-growing bacterial cells in the centre, and fragmentation which leads to cell detachment and spread of infection.

As reported before, the number of infections associated with antibiotic-resistant bacteria is continuously increasing. Microorganisms growing in biofilms cause many of these infections. The most common biofilm-forming bacteria associated with human infections are: *E. faecalis*, *S. aureus*, *S. epidermidis*, *Streptococcus viridans*, *E. coli*, *K. pneumoniae*, *Moraxella catarrhalis*, *Proteus mirabilis* and *P. aeruginosa* [15]. Biofilms may be one of the leading causes for a shift from acute-phase diseases to chronic diseases. Most common diseases involving bacteria able to form biofilm are biliary tract infections, cystic fibrosis, dental caries, endocarditis, otitis and periodontal diseases. Moreover, several infections may be associated with foreign body material such as contact lens, sutures, artificial heart valves, arteriovenous shunts, catheters and orthopedic prostheses. The sites of infections may be different but the characteristics (mechanism for biofilm formation and development of resistance) of the causative agent are similar. Biofilms are communities of microorganisms attached to a solid surface. These adherent cells are frequently embedded within a self-produced matrix of extracellular polymeric substance. Biofilm extracellular polymeric substance is a conglomeration composed of DNA, proteins and polysaccharides [73]. The matrix is produced under the control of enzymes secreted in response to nutrient availability [74]. Biofilms develop in natural aquatic systems, water pipes, on the teeth, on medical devices [15]. The signals that promote biofilm rapid formation are: (i) presence of a suitable surface; (ii) increase of extracellular iron; (iii) presence of indole, polyamines, calcium and bile salts [75,76,77]. In the initial phase of biofilm formation, bacterial attacks proliferate, forming microcolonies and attracting surrounding cells. The mature biofilm is a real microbial community that exchanges and shares products in a dynamic manner [78]. In fact, cell growth, death, nutrients acquisition, the accumulation of waste products, mechanisms of motility and exopolysaccharide synthesis can affect the structure and attributes of biofilms [77,79]. In Figure 3 is represented a biofilm formation divided into the following phases: (i) the planktonic form, in which the separated cells are floating or swimming independently in a liquid support; (ii) the aggregated state, or sessile, in which cells are closely bound and firmly attached to one another and also, usually, to a solid surface.

The change in behaviour is triggered by a chemical communication mechanism that differs between species. Some species, for example, can produce acylhomoserine lactones as a “rest” signal, which induces planktonic cells that surround the phenotypic variation to change into the sessile state, through a different expression of the genes of the cell. As the understanding of biofilm increases, it is becoming evident that biofilm phenotypes cannot be analysed and eventually fought using the traditional principles of bacteriology. In fact, the properties of a biofilm are similar to the properties of a polymer and not to the properties of a sum of cells. Indeed, biofilms possess elastic and viscous properties which allows the community to adhere, grow in a tridimensional structure and move inside the lumen of a catheter or a similar device. The pathogenicity of biofilms can be summarised by the following properties: (i) attachment to solid surfaces to high density; (ii) increased metabolic efficiency of the community; (iii) evasion of host-defences; (iv) horizontal gene transfer; (v) antimicrobial resistance; (vi) detachment of microbial aggregates able to colonise other sites. Bacterial biofilms are particularly unmanageable by antibiotic treatments not only due to an increase in transmission of resistance markers within the biofilm community, but also because the extracellular matrix hampers antibiotic diffusion, because the effectiveness of antibiotics is inactivated more easily, and because metabolically inactive persistent cells survive treatment. Together these features make bacterial biofilms up to 1000 times more resistant to antibiotics than planktonic cells. The antibiofilm activity of AgNPs has been demonstrated in a number of studies and is briefly described in the rest of the section. One pioneering study was performed to analyse the interactions of AgNPs with *Pseudomonas putida* biofilms. The results suggested that biofilms are impacted by the treatment with AgNPs. The nanoparticles analyzed in the study were of quite large dimensions (over 60 nm) [80]. One of the first reports on the antibiotic effect of AgNPs on *P. aeruginosa* and *S. epidermidis*, and their effect on biofilm formation, was produced by Kalishwaralal *et al.* [81]. The study focused on two important pathogens causing keratitis and the effect of a 2 h of treatment with AgNPs at a concentration of 100 μM showed that a 95% and 98% decrease of the biofilm was obtained. Therefore the authors concluded that AgNPs are able to induce the detachment of *P. aeruginosa* and *S. epidermidis* with rapidity and efficiency, opening clinical possibilities of alternative therapies. An important feature to evaluate the real efficiency of the nanoparticles is derived from the chosen stabilization method employed. To this regard several coatings and chemicals have been reported: (i) starch was successfully employed to prepare AgNPs which had a disrupting effect on biofilms produced by *P. aeruginosa* and *S. aureus* [82]; (ii) citrate-capped AgNPs of various sizes were shown to inhibit *P. aeruginosa* PAO1 biofilms [83]; (iii) polyvinylpyrrolidone (PVP) showed good antibacterial activity towards *S. aureus*, *E. coli*, *P. aeruginosa*, *Bacillus subtilis*, and good fungicidal activity against various yeasts and molds [84]; (iv) β-cyclodextrin is also an effective capping and stabilizing agent that reduces the toxicity of AgNPs against the mammalian cell while enhancing their antibiofilm activity [85]. Mohanty *et al.* [82] used a simple and environment friendly approach to form stable colloids of nontoxic AgNPs using starch to reduce silver nitrate to silver metal and simultaneously stabilize the nanoparticles in starch solution. Then they tested the effect of AgNPs on biofilm formation by *P. aeruginosa* and *S. aureus* with varying concentrations of AgNPs. Longer treatments (48 h) increased the antibiofilm efficiency to approximately 65% and 88% reduction in biofilm formation at micromolar concentrations. The ability to disrupt *P. aeruginosa* biofilm formation after treatment with the antimicrobial peptide LL-37, already known to impair biofilm formation, and AgNPs was also analysed and, in comparison to LL-37, treatment with AgNPs resulted in a 3-fold reduction of biofilm formation. Multidrug resistant (MDR) strains of *P. aeruginosa* were treated with AgNPs to investigate the eventual increased resistance compared to sensible strains. In the multidrug resistant strains, the inhibition rate of AgNPs was highest at concentration of 20 μg/mL similarly to the parental strain, therefore biofilms derived from multiresistant bacteria do no show an increased resistance to silver [69].

Antibiofilm action of AgNPs of 8.3 nm in diameter stabilized by hydrolyzed casein peptides on Gram-negative bacteria (*E. coli*, *P. aeruginosa* and *Serratia proteamaculans*) was investigated by Radzig *et al.* [86]. A strong inhibition of biofilms formation was observed. Interestingly, several *E. coli* strains with mutations in genes responsible for the repair of DNA containing oxidative lesions (mutY, mutS, mutM, mutT, nth) were also analyzed and found less resistant to AgNPs than wild type strains, suggesting a possible involvement of these genes in repair of AgNP-produced damages to cellular DNA. The outer membrane of Gram-negative contains water-filled channels (also called porins) to allow the exchange with the environment of low-molecular weight compounds. Porins are involved in the transport of Ag-ions and *E. coli* bacteria expressing mutated porin proteins are less susceptible to silver ions action [87]. Radzig *et al.* [86] confirmed that *E. coli* mutant strains deficient in OmpF or OmpC porins were 4–8 times more resistant to AgNP when compared to the wild type strain, suggesting that porins have a key role in allowing AgNPs to exert their antibacterial effect.

The anti-biofilm activity of silver nanoparticles was also demonstrated in other studies mainly focused on bacteria showing resistance to conventional antibiotics [88,89]. The biofilm formation by methicillin resistant *S. aureus* (MRSA) and methicillin resistant *S. epidermidis* (MRSE) isolated from infected wounds was also analyzed by confocal laser scanning microscopy (CLSM) techniques which provided concrete evidence of the ability of AgNPs to block bacterial growth and to prevent the glycocalyx formation. A complete anti-biofilm activity was obtained with AgNPs at a concentration lower than 50 μg/mL [88]. Gurunathan *et al.* [90] analyzed the antibacterial and antibiofilm activity of antibiotics or AgNPs, or combinations of both against *P. aeruginosa*, *Shigella flexneri*, *S. aureus*, and *Streptococcus pneumonia*. They were able to show a clear enhancing effect for ampicillin and vancomycin against either Gram-negative or Gram-positive bacteria, suggesting that combining AgNPs with antibiotics could be a possible alternative therapeutic strategy against bacterial infectious diseases.

An interesting evolution of using nanoparticles against bacterial biofilms is represented by silver-coated magnetic nanoparticles, in fact, engineered multimodal nanoparticles comprising a magnetic core and a silver ring showed promising results [91]. Along this line, magnetic and antibacterial properties have been exploited by creating superparamagnetic iron oxide nanoparticles (SPION) conjugated with silver to demonstrate that MRSA biofilms can be eradicated without the need of antibiotics. MRSA biofilms treated with 1 mg/mL of silver-conjugated SPION resulted in a consistent mass decrease. Moreover, SPION anti-biofilm efficacy is further improved in the presence of an external magnetic field.

Silver is nowadays used on medical devices to support anti-biofilm activity. Biofilms from clinical isolates of *P. aeruginosa* were treated with gum arabic capped-silver nanoparticles (GA-AgNPs) showing a concentration dependent inhibition of bacterial growth and treatment of catheters with GA-AgNPs at 50 µg/mL resulted in 95% inhibition of bacterial colonization of the plastic catheter surface [88].

Other authors have also shown the applications of nanosilver as antibiofilms for coating catheters [92,93,94] with positive results against both Gram-positive and Gram-negative bacteria. Furthermore, no significant accumulation of silver was detected in the main organs of the test animals in which engineered catheters had been implanted [92]. Dental applications were obtained with composites containing silver nanoparticles that can act against *S. mutans* biofilm [95]. Also, bone cements modified with AgNPs significantly reduced biofilm formation on the surface of the cement [96]. Some medical devices, as well as surgical masks [97], coated with AgNPs are already in clinical trials [93] with promising results [98,99]. Furthermore, recent studies suggest the use of wound dressings treated with AgNPs to prevent or reduce microbial growth in wounds and to improve the outcomes of healing [100]. A bioactive chitosan hydrogel membrane including AgNPs showed a synergistic activity of chitosan and AgNPs to reduce the growth of *S. aureus*, *E. coli*, *S. epidermidis*, *P. aeruginosa* strains and to disrupt mature biofilms [101].

## 4. Conclusions

The potential benefits of nanotechnology in biomedical and industrial applications have become widely accepted and are the most promising sector for the generation of new applications in medicine. It is now clear that AgNPs possess a strong antibacterial and antiviral activity, highlighted by several studies. AgNPs have the ability to interact with various microorganisms (such as bacteria) and also impact both the growth of and mature bacterial biofilms and, therefore, could be used as broad spectrum antimicrobials. The antibacterial effect appears to be conferred by their ultrasmall size and increased surface area, through which they destroy the membrane, cross the body of the microbe and create intracellular damage. Due to the structural difference in the composition of the cell walls of Gram-positive and Gram-negative AgNPs have significantly less effect on the growth of Gram-positive bacteria. The Gram-negative bacteria have a layer of lipopolysaccharides on the outside, and present below a thin (7 to 8 nanometers) layer of peptidoglycan. Although lipopolysaccharides are composed of lipids covalently bound to polysaccharides, there is a lack of rigidity of the overall structural envelope. The negative charges on the lipopolysaccharides are attracted to the weak positive charge of AgNPs. On the other hand, the cell wall of Gram-positive bacteria is mainly composed of a thick layer (20 to 80 nanometers) of peptidoglycan consisting of linear polysaccharidic chains cross-linked by short peptides to form a three-dimensional rigid structure. The stiffness and the extensive cross-linking not only reduce the bacterial cell wall anchoring sites for AgNPs but also render the wall itself more difficult to penetrate. However, the same features that make AgNPs attractive, at the same time raise important issues such as the toxicity and environmental safety. AgNPs’ antibacterial effects have been described in detail, but their mechanism of action is still unclear. A multifaceted mechanism against microorganisms seem to be due to nanoparticle interactions with the bacterial surfaces, as well as to their particular structure. Defining AgNPs’ mechanism of action is, nowadays, a priority for biomedical research and more research on the bioactivity and biocompatibility of AgNPs is necessary. Understanding the kinetics of dissolution that lead to transformations of AgNPs in the presence of specific inorganic ligands is crucial to determining their antimicrobial activity and overall toxicity in the environment. Silver ions (Ag^+^), released by AgNPs, are likely to interact with chloride (Cl^−^) which is often present in bacterial growth media and exhibits a strong affinity for oxidized silver. High concentrations of chloride ions in the routinely used media can cause precipitation of Ag ions as AgCl, thus masking the contribution of dissolved silver to AgNPs antibacterial effect. This consideration should influence the choice of the medium to be used when evaluating antimicrobial effects and more studies are needed to investigate the contribution of AgCl to the observed antibacterial activity of AgNPs. The studies on the combined use of AgNPs with other antimicrobial agents can help reduce the problem of toxicity and to avoid the potential for development of resistance and, above all, strongly enhance the microbicidal effect. The broad spectrum of bioactivity of AgNPs makes them promising agents not only to fight infections, but in many other biomedical areas. In Table 3 we include some ongoing clinical trials.

**Table 3:** AgNP clinical trials.

ClinicalTrials.gov Identifier	Status	Study
NCT00341354	Completed	Coated Endotracheal Tube and Mucus Shaver to Prevent Hospital-Acquired Infections.
NCT00659204	Unknown	Efficacy of AgNp Gel Versus a Common Antibacterial Hand Gel.
NCT00965198	Completed	Comparison of Infection Rates Among Patients Using Two Catheter Access Devices.
NCT01048112	Unknown	*Campylobacter jejuni* Challenge Model Development: Assessment of Homologous Protection.
NCT01258270	Completed	Efficacy and Patient Satisfaction With AQUACEL^®^ Ag Surgical Dressing Compared to Standard Surgical Dressing.
NCT01598480	Completed	To Study the Healing Effect of Silver Impregnated Activated Carbon Fiber Wound Dressing on Superficial Dermal Burn.
NCT01598493	Completed	To Study the Healing Effect of Silver Impregnated Activated Carbon Fiber Wound Dressing on Deep Dermal Burn.
NCT01821664	Not yet recruiting	Vascular Graft Infections.
NCT02099240	Not yet recruiting	Patients Response to Early Switch To Oral: Osteomyelitis Study.
NCT02116010	Not yet recruiting	Evaluation of Phage Therapy for the Treatment of *E. Coli* and *P. Aeruginosa* Wound Infections in Burned Patients.
NCT02213237	Recruiting	The Application of SERS and Metabolomics in Sepsis.
NCT02225158	Recruiting	Immune Responses to *Mycobacterium Tuberculosis* (Mtb) in People With Latent Tuberculosis Infection With or Without Concomitant Helminth Infection.
NCT02241005	Recruiting	Theraworx Bath Wipes Versus Standard Bath Wipes in the Reduction of Vancomycin-Resistant Enterococci.
NCT02277171	Not yet recruiting	Evaluation of Safety and Tolerability of Nitric Oxide Impregnated Urinary Catheters.
